# A novel cross-species differential tumor classification method based on exosome-derived microRNA biomarkers established by human-dog lymphoid and mammary tumor cell lines’ transcription profiles

**DOI:** 10.14202/vetworld.2022.1163-1170

**Published:** 2022-05-11

**Authors:** Kaj Chokeshaiusaha, Thanida Sananmuang, Denis Puthier, Catherine Nguyen

**Affiliations:** 1 Department of Veterinary Science, Faculty of Veterinary Medicine, Rajamangala University of Technology Tawan-OK, Chon Buri, Thailand; 2 Aix-Marseille University, INSERM UMR 1090, TAGC, Marseille, France

**Keywords:** exosome-derived microRNA, meta-analysis, ortholog, support vector machine, tumor

## Abstract

**Background and Aim:**

Exosome-derived microRNA (miRNA) has been widely studied as a non-invasive candidate biomarker for tumor diagnosis in humans and dogs. Its application, however, was primarily focused on intraspecies usage for individual tumor type diagnosis. This study aimed to gain insight into its application as a cross-species differential tumor diagnostic tool; we demonstrated the process of identifying and using exosome-derived miRNA as biomarkers for the classification of lymphoid and mammary tumor cell lines in humans and dogs.

**Materials and Methods:**

Exosome-derived miRNA sequencing data from B-cell lymphoid tumor cell lines (n=13), mammary tumor cell lines (n=8), and normal mammary epithelium cultures (n=4) were pre-processed in humans and dogs. F-test and rank product (RP) analyses were used to select candidate miRNA orthologs for tumor cell line classification. The classification was carried out using an optimized support vector machine (SVM) with various kernel classifiers, including linear SVM, polynomial SVM, and radial basis function SVM. The receiver operating characteristic and precision-recall curves were used to assess the performance of all models.

**Results:**

MIR10B, MIR21, and MIR30E were chosen as the candidate orthologs from a total of 236 human-dog miRNA orthologs (p≤0.01, F-test score ≥10, and RP score ≤10). Their use of polynomial SVM provided the best performance in classifying samples from various tumor cell lines and normal epithelial culture.

**Conclusion:**

The study successfully demonstrated a method for identifying and utilizing candidate human-dog exosome-derived miRNA orthologs for differential tumor cell line classification. Such findings shed light on a novel non-invasive tumor diagnostic tool that could be used in both human and veterinary medicine in the future.

## Introduction

The oncogenic nature of human and canine tumors was strikingly similar [1]. With such cognate characteristics, the cross-species tumor analysis provided a valuable insight into the novel primitive features important for diagnosis and therapy. Lymphoid tumors of B-cell origin and mammary tumors in humans and dogs were well-recognized for their mutual characteristics [2]. Because these tumors had a high incidence of malignancy, their genetic biomarkers were extensively studied in various tumor environments [1,3]. The exosome, a membrane-bound extracellular vesicle secreted by cells, has recently been regarded as a promising landmark in the discovery of novel biomarkers for such tumor types [4-6].

Exosomes are typically released by a variety of cell types. They were derived from the intracellular endosome and contained a variety of mediators for distant cell communication and regulation [7,8]. MicroRNA ([miRNA], also known as small non-coding RNA (18-22 nucleotides), is a type of exosome mediator that is responsible for the cell’s negative gene expression through the post-transcriptional degradation of messenger RNA (mRNA) [9]. Exosome-derived miRNA dysregulation was found to be common in lymphoid and mammary tumors [4-7]. These disordered miRNAs were transferred and modulated the gene transcription profiles of various distant cell types during their oncogenesis, promoting tumor progression, and metastasis [7,10].

Several studies found a strong correlation between changes in certain exosome-derived miRNA levels and lymphoid and mammary tumor malignancies [3-6,11]. Changes in the expression levels of several exosome-derived miRNA orthologs were found to be correlated between human and canine mammary tumors [4,10] and lymphoid tumor cell lines [11,12]. In line with such *in vitro* evidence, several miRNA biomarkers were found to be altered in the blood of patients with the corresponding tumor types [6,12,13]. These related shreds of evidence supported the advantages of exosome-derived miRNA research in the tumor cell lines to provide insight into candidate peripheral miRNA markers for clinical tumor diagnosis [6,12].

Exosome-derived miRNAs were found to be valuable biomarkers in human and canine oncology [4,6,11,12]. Despite this potential, their studies were primarily focused on intraspecies implementation with tumor specificity. This approach, however, contradicted clinical practice, which states that different tumor types with similar clinical or pathological characteristics should be distinguished from one another [14-16]. As a result, rather than only being able to distinguish patients with specific tumor types from the healthy ones, the identified miRNA biomarker should suffice for such an objective. Furthermore, biomarkers that can be used across species should provide us with extensive information about their universal roles in the oncogenesis of each specific tumor type.

Sequencing technology has been widely used in canine and human exosome-derived miRNA profiling [4,10,12]. Despite the limited evidence from the direct comparison study of canine and human tumor exosome-derived miRNA profiles, their available datasets in the database allowed for meta-analysis among them. It should also be noted that most intraspecies exosome-derived miRNA studies in tumors interpret statistical changes in target miRNA expression levels. While such resolution was useful for distinguishing individual tumor samples from normal ones, its rigid interpretation would limit efficiency in cross-species differential tumor diagnosis, which requires different tumor types obtained from more than 1 species to be classified as different classes.

This study aimed to attain deeper knowledge about exosome-derived miRNA application as the tool for cross-species differential tumor diagnosis in humans and dogs. In this study, the human-dog exosome-derived miRNA transcription profiling meta-analysis of lymphoid and mammary tumor cell lines acquired from both species was performed to establish a method for cross-species differential tumor classification by exosome-derived miRNA biomarkers. The tumor cell line type classification was successfully archived in both species with the proper optimization.

## Materials and Methods

### Ethical approval

All datasets used in this study were available on NCBI sequence read archive (SRA) public site (https://www.ncbi.nlm. nih.gov/sra), and no ethical approval was required.

### Study period and location

The study was conducted from April to October 2021. The human-dog exosome-derived miRNA transcription profiling meta-analysis was conducted at the Faculty of Veterinary Medicine, Rajamangala University of Technology Tawan-OK, Thailand.

### Exosome-derived miRNA data

The sequence read archive database was used to retrieve small RNA sequencing data derived from exosomes in the culture medium of normal mammary epithelium, mammary tumor cell lines, and various lymphoid tumor cell lines from both humans and dogs which were retrieved from the SRA database (https://www.ncbi.nlm. nih.gov/sra) [4,6,11,17,18]. All samples of canine and human lymphoid tumor cell lines were B-cell origin. Except for canine mammary tumor cell lines, which were generated from biopsies obtained from canine mammary tumor cases, most cell lines, including human mammary tumor cell lines, were commercially available (Table-1).

**Table 1:** Exosome-derived RNA datasets.

Dataset	Cell type	Source of exosome
SRR7505863	Canine mammary tumor	Mammary tumor cell line from biopsy specimen
SRR7505858	Canine mammary tumor	Mammary tumor cell line from biopsy specimen
SRR7505859	Canine mammary tumor	Mammary tumor cell line from biopsy specimen
SRR7505862	Canine mammary tumor	Mammary tumor cell line from biopsy specimen
DRR127938	Canine lymphoid tumor	CLBL-1 cell line
DRR127939	Canine lymphoid tumor	CLBL-1 cell line
DRR127942	Canine lymphoid tumor	GL-1 cell line
DRR127943	Canine lymphoid tumor	GL-1 cell line
SRR7505860	Canine epithelium	Normal mammary epithelial cells
SRR7505865	Canine epithelium	Normal mammary epithelial cells
SRR3713945	Human mammary tumor	MDA-MB-231 cell line
SRR3713946	Human mammary tumor	MDA-MB-231 cell line
SRR3713943	Human mammary tumor	MCF-7 cell line
SRR3713944	Human mammary tumor	MCF-7 cell line
SRR1563017	Human lymphoid tumor	BJAB cell line
SRR1563060	Human lymphoid tumor	IK140508 cell line
SRR1563062	Human lymphoid tumor	IM-1 cell line
DRR127191	Human lymphoid tumor	Mutu- cell line
DRR127193	Human lymphoid tumor	Mutu-1 cell line
DRR127195	Human lymphoid tumor	Mutu-3 cell line
SRR1563058	Human lymphoid tumor	Mutu-1 clone 9 cell line
SRR1563056	Human lymphoid tumor	Mutu-5 cell line
SRR1563064	Human lymphoid tumor	RN cell line
SRR3713941	Human epithelium	Normal mammary epithelial cells
SRR3713942	Human epithelium	Normal mammary epithelial cells

### Data pre-processing

All exosome-derived RNA sequencing datasets (Table-1) were assessed for quality, aligned with their corresponding genome assembly (GRCh38 for humans and CanFam3.1 for dogs), and counted for each annotated miRNA ortholog (CanFam3.1 Ensembl gene annotation) using a method similar that described previously [19]. For consistency, human gene symbols were used to refer to all orthologs in this study. In brief, the genome assembly and annotated nucleotide sequences of both humans and dogs were obtained from the Ensembl database (https://asia.ensembl.org/info/data/ftp/index.html). The “Flexbar 3.0” software archived the adapter trimming and quality trimming processes [20,21]. The sequences chosen for genome alignment were 18-30 nucleotides long, with Phred scores ≥30 for at least 50% of the bases. The selected sequences were aligned and counted using the “STAR” aligner [22], and the suitable genome assembly – CanFam3.1 and GRCh38 for canine and human sequences, respectively. With batch correction by the Combat-Seq method, all sequences aligned to annotated miRNA orthologs would be included in the pooled human-dog miRNA library [23]. Finally, the pooled library was normalized using the Transcripts Per Million metric followed by log2 transformation.

### Candidate exosome-derived miRNA selection

The principle presented in our previous studies and was used to select candidate miRNA orthologs for classifying lymphoid and mammary tumor cell lines [24,25]. The miRNA orthologs were differentially expressed among lymphoid tumor cell lines, mammary tumor cell lines, and normal epithelium cell cultures, which were identified using F-test results (acquired from 1000 learning datasets generated through 3-fold cross-validation) and the “CMA” package [26]. Differentially expressed miRNA orthologs with an importance value ≥10 would be further validated using the Rank Product (RP) analysis (p≤0.01) and the “RankProd” package [27]. Because this study aimed to identify the exosome-derived miRNA candidates with high potential to classify different tumor cell lines, only miRNA orthologs with high-ranking expression orders from RP analysis (RP score ≤0) would be considered due to their significant differences in expression levels.

### Tumor cell line classification performance of support vector machine (SVM)

SVM with different kernel classifiers are linear, polynomial, and radial basis function (Rbf). Classifiers were trained and optimized by the “Optunity” and “Scikit-learn” packages (3-fold cross-validation, 1000 iterations) to determine the performance of the candidate miRNA orthologs in tumor cell line classification [28,29]. The receiver operating characteristic (ROC) and precision-recall (PR) curves would be used to compare the performances of the optimized models obtained from all kernels – linear SVM, polynomial SVM, and Rbf SVM. The ROC curve would assess the model by determining the relationship between true-positive values versus false-positive values using all available classification thresholds. The acquired area under the ROC curve (AUC) summarized each model’s aggregate performance across all thresholds; the higher the AUC, the better balanced the model. On the other hand, the PR curve would determine the trade-off between PR for all available classification thresholds. The balanced model was determined by the collective maintenance of high precision values as recall values changed.

### Data visualization

All heatmaps were created using the “ComplexHeatmap” package [30]. The barplot, ROC curves, and PR curves were plotted using the “Scikit-learn” [29] and the “Matplotlib” packages, respectively [31].

## Results

### Expressions of human-dog exosome-derived miRNA orthologs among cell cultures

**Figure-1:**
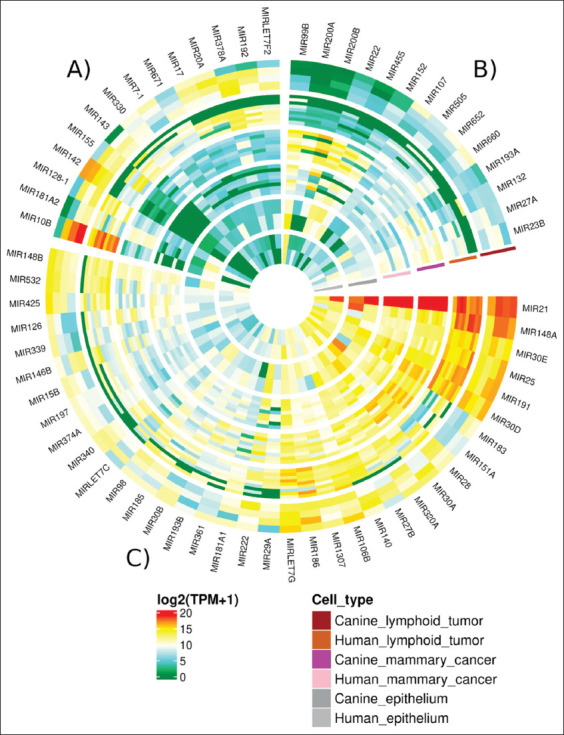
The circular heatmap illustrated the top 50 most abundant exosome-derived miRNA orthologs expressed by mammary and lymphoid tumor cell lines. The heatmap was divided into three sections. Sections A and B contained the abundant miRNA orthologs regarded as top expressed miRNAs only in lymphoid tumor and mammary tumor cell lines, respectively. On the other hand, section C contained abundant miRNA orthologs coexpressed between lymphoid and mammary tumors. Colors in log2 (TPM+1) indicated the miRNA expression levels ranking from highest (red) to lowest (green) among different human and canine cell cultures indicated by the “Cell type” legend.

Two hundred thirty six human-dog miRNA orthologs were discovered (CanFam3.1 Ensembl gene annotation). The top 50 most abundant exosome-derived miRNAs expressed by lymphoid and mammary tumor cell lines were determined by calculating average expression levels (Figure-1). Among them, 36 miRNA orthologs were found to be mutually abundant between tumor types, with the most notably expressed miRNA orthologs – MIR21, MIR148A, MIR30E, MIR25, MIR191, and MIR30D being presented (section C of the circular heatmap in Figure-1). The number of uniquely abundant orthologs expressed by each tumor type, on the other hand, was 14 orthologs (sections A and B of the circular heatmap in Figure-1). In line with previous findings, among the abundant exosome-derived miRNAs secreted by lymphoid tumor cell lines, MIR143, MIRLET7F2, MIRLET7G2, and MIR30D were identified as abundant miRNAs expressed by lymphoid tumor cell lines (Figure-1) [11,18]. Similarly, several exosome-derived miRNA orthologs previously reported for increased expression in both canine and human mammary tumor cell lines, including MIR21, MIR106B, MIR181A1, MIR183, MIR200B, and MIRLET7G, were presented [4].

### Selection of candidate exosome-derived miRNA orthologs for tumor cell line classification

**Figure-2:**
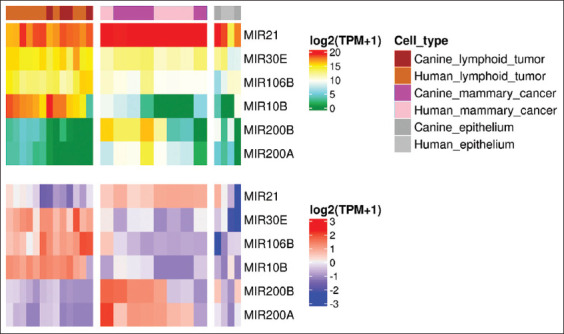
Differentially expressed exosome-derived miRNA orthologs among mammary tumor cell line cultures, lymphoid tumor cell line cultures, and normal epithelium cell cultures were demonstrated by non-scaled heatmap (upper) and scaled heatmap (lower), accordingly.

**Figure-3:**
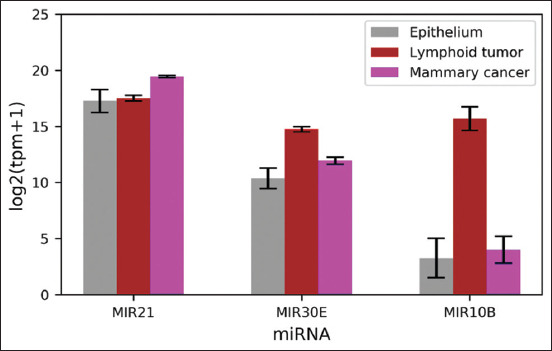
Expression levels of FiMIR10B, MIR21, and MIR30E in each cell type. These miRNAs were selected as candidates for support vector machine classifiers.

The process as described in the methodology was used to select the candidate exosome-derived miRNA orthologs. A total of six miRNA orthologs – MIR21, MIR30E, MIR106B, MIR10B, MIR200A, and MIR200B were identified as differentially expressed orthologs among mammary tumor cell lines, lymphoid tumor cell lines, and normal epithelial cell cultures (importance ≥10 and p≤0.01) (Figure-2). Only MIR10B, MIR21, and MIR30E inherited RP scores of <10 and were thus chosen as candidate orthologs for further tumor classification analysis using the SVM model. MIR21 was found to be the most abundant in mammary tumor cell culture, while MIR10B and MIR30E were found to be the most abundant in lymphoid tumor cell culture (Figure-3).

### Tumor classification performance of SVM models based on expressions of MIR10B, MIR21, and MIR30E

**Figure-4:**
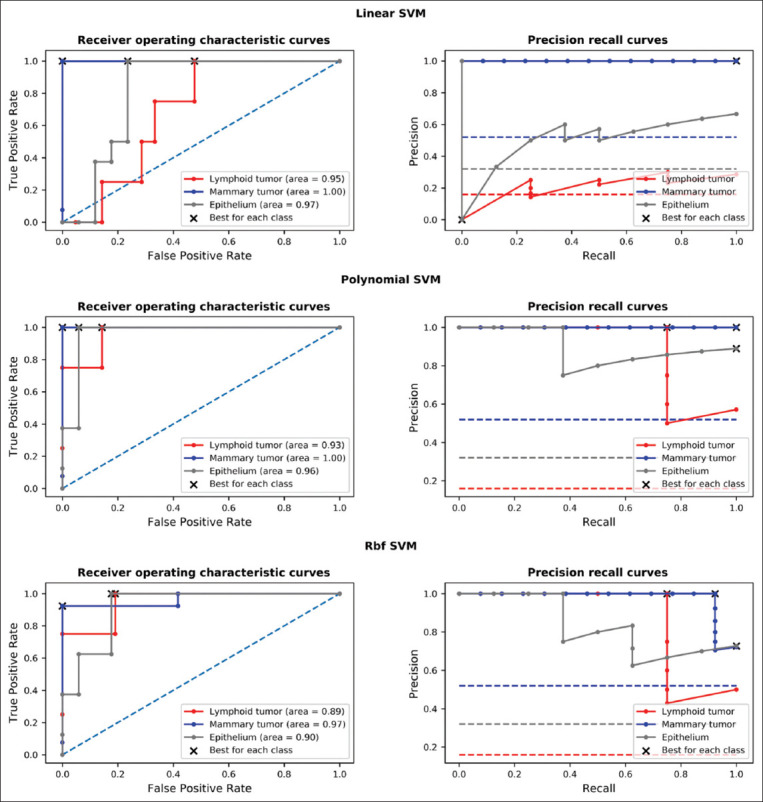
The performances of optimized linear support vector machine (SVM), polynomial SVM, and Rbf SVM models in classifying mammary tumor cell line, lymphoid tumor cell line, and normal epithelium cell cultures utilizing MIR10B, MIR21, and MIR30E as variables were demonstrated. The receiver operating characteristic and precision-recall curves of each sample class – lymphoid tumor (red), mammary tumor (blue), and epithelium (gray) were drawn with their corresponding optimal thresholds (most balanced) marked by the black crosses. The dash lines indicated the classifying performance of each cell class without the model.

As described in the methodology, SVM with linear kernel (linear SVM), polynomial kernel (polynomial SVM), and Rbf kernel (Rbf SVM) classifiers were optimized for their parameters based on expression values of MIR10B, MIR21, and MIR30E. Table-2 shows the optimized parameters of each model that was obtained. The classification performance of each model was demonstrated using ROC and PR curves, which displayed different results on different sample classes (lymphoid tumor, mammary tumor, and epithelium lines in Figure-4). This result showed that polynomial SVM was the most well-balanced model. This was demonstrated by the maximum AUCs and the lowest PR trade-off obtained from each sample class when compared to linear SVM and Rbf SVM (interpretation as described in the methodology).

**Table 2:** SVM models and their optimized parameters.

Model	Abbrev.	Optimized parameters
Support vector machine with linear kernel	Linear SVM	^c^ C=(1.5087890625)
Support vector machine with polynomial kernel	Polynomial SVM	^c^ C=(1.82436524043723), ^e^ coef0=(0.399464647761237), ^f^ degree=(2.87494140625)
Support vector machine with radial basis function kernel	Rbf SVM	^c^ C=(5.09560973685087) ^d^ gamma=(-2.84106941646877)

^a^ coef=Coefficients for the linear regression, ^b^ inter=Intercept for linear regression,

c C=Regularization parameter,

d gamma=Kernel coefficient,

e coef0=Independent term,

f degree=Degree of the polynomial kernel function, SVM=Support vector machine

## Discussion

This research demonstrated a novel method for identifying and using human-dog exosome-derived miRNA biomarkers for lymphoid and mammary tumor cell line classification. Based on the SVM model, this study, as far as we know, established the first cross-species method for different tumor type classification using miRNA biomarkers and an optimized SVM model. Despite the successful demonstration, some concerns should be raised as critical limitations for implying the knowledge gained from this research. This included a lack of normal lymphocyte culture as a suitable control group for the lymphoid tumor cell line. The scarcity of tumor cell line sources also raised concerns about generalizing the use of the identified markers to other lymphoid and mammary tumor cell line types not included in this study. In similar situations, the generalization of acquired knowledge for clinical practice must be considered. Since the purpose of this study was only to introduce the concept of universal exosome-derived miRNA biomarkers using cell culture sequencing data, further clinical application of the knowledge required further validation of both markers and models with proper miRNA sequencing data such as those obtained from tumor patients’ peripheral exosomes.

Exosome-derived miRNA orthologs were abundantly expressed in lymphoid and mammary tumor cell lines from humans and dogs (Figure-1). Furthermore, several of them replicated those previously reported in other studies [4,10,11,18], thereby validating the pooled cross-species datasets library. It should be noted that this study will only look at candidate miRNAs that have a significant impact on tumor classification model. As a result, only candidate miRNAs with such significance would be exclusively discussed in this study.

This study aimed to develop a universal model for using miRNA orthologs in the future diagnosis of human and veterinary oncology. In clinical practice, plasma or serum was most likely used as a sample source for determining exosome-derived miRNA. Given that the normal circulatory system produces a variety of miRNAs, such a baseline should influence the sensitivity of candidate miRNA orthologs, particularly those referred to from the closed system model, such as exosome-derived miRNA in cell culture medium. As a result, the miRNA orthologs with the highest fold changes in expression levels across cell culture types – MIR21, MIR30E, and MIR10B were more preferable (RP score ≤10) (Figure-2).

MIR21, MIR30E, and MIR10B were found to be important in lymphoid and mammary tumor diagnosis. Exosome-derived MIR21, a well-established tumor-promoting miRNA, has been shown to suppress several tumor suppressor gene expressions in distant cells [8,10]. High circulatory MIR21 levels have also been linked to poor prognoses in patients with B lymphoma and mammary tumors [6,13]. MIR30E and MIR10B, on the other hand, were members of miRNA families with varying regulatory roles in disease development. While the regulatory roles of both miRNAs in lymphoid and mammary tumors remain unknown, their presence in circulation was linked to tumor incidence. It has been reported that patients with classical Hodgkin lymphoma have a high level of MIR30E in their blood [32]. Similarly, high MIR10B levels have been found in patients with diffuse large B-cell lymphoma and mammary tumors [12,33].

Without prior knowledge about the relationship among target miRNAs considered in the categorization process, the SVM was regarded as one of the most feasible classifying models for categorization [34,35]. SVM was used in this study because of its ability to deal with the unknown interconnection between the candidate miRNA orthologs in the various cell type samples presented in this study. Despite the fact that polynomial SVM was identified as the most balanced model, different threshold adjustments were still required for the best results of each tumor cell type classification. This notified the need to fine tune the threshold for the best diagnostic result of each tumor type. However, it should be noted that the model in this study was optimized using the cell cultures presented in this study (Table-2). All SVM models should be reoptimized for the update with the inclusion of more samples, different sample types, and/or clinical samples collected from real cases.

## Conclusion

In this study, a novel method for human-dog tumor classification using the SVM classifier model with candidate exosome-derived miRNA orthologs was successfully demonstrated, and it is regarded as an initial model for clinical validation. It is worth noting that a similar concept could be applied in other research fields, such as infectious diseases, autoimmune diseases, and metabolic diseases, providing an alternative approach for disease diagnosis based on certain exosome-derived miRNA expressions.

## Authors’ Contributions

KC and TS: Study design, analyzed data, and drafted the manuscript. KC: Collected the data. DP and CN: Reviewed the manuscript. KC: Carried out technical coding correction. All authors have read and approved the final manuscript.
